# Procoagulant Status and Fibrinolytic Activity in COVID-19 Patients during Illness and Convalescence

**DOI:** 10.3390/biomedicines12010042

**Published:** 2023-12-22

**Authors:** Olga S. Beznoshchenco, Andrey Yu. Romanov, Nataliya V. Dolgushina, Elena A. Gorodnova, Tatiana Yu. Ivanets, Ekaterina L. Yarotskaya, Aleksey V. Pyregov, Sergej V. Grachev, Gennady T. Sukhikh

**Affiliations:** 1 National Medical Research Center for Obstetrics, Gynecology and Perinatology Named after Academician V.I. Kulakov of the Ministry of Health of the Russian Federation, 117997 Moscow, Russia; o_beznoshchenko@oparina4.ru (O.S.B.); n_dolgushina@oparina4.ru (N.V.D.); e_gorodnova@oparina4.ru (E.A.G.); t_ivanets@oparina4.ru (T.Y.I.); e_yarotskaya@oparina4.ru (E.L.Y.); a_pyregov@oparina4.ru (A.V.P.); g_sukhikh@oparina4.ru (G.T.S.); 2 Federal State Autonomous Educational Institution of Higher Education I.M. Sechenov First Moscow State Medical University of the Ministry of Health of the Russian Federation (Sechenov University), Ministry of Health of the Russian Federation, 119048 Moscow, Russia; grachevscience@gmail.com

**Keywords:** COVID-19, von Willebrand factor, ADAMTS-13, hypercoagulation, thrombosis

## Abstract

SARS-CoV-2 (Severe Acute Respiratory Syndrome-related CoronaVirus 2) activates the immune system, causing thrombin dysregulation and tissue damage and reduces endothelium anticoagulant function, leading to excessive thrombin formation. Hypercoagulability, which causes multiple organ failure in critically ill COVID-19 (COronaVIrus Disease 2019) patients, can be detected by viscoelastic tests like thromboelastography and rotational thromboelastometry (ROTEM). We aimed to assess the coagulation system status and fibrinolytic activity using ROTEM thromboelastometry in patients with COVID-19 and convalescents. The observational prospective study included 141 patients with COVID-19: Group 1—patients with mild (*n* = 39), Group 2—patients with moderate (*n* = 65), and Group 3—patients with severe (*n* = 37) COVID-19. The coagulation status was assessed twice—during the disease and in convalescence. The male gender, age > 56 years, overweight, and obesity were risk factors for developing severe COVID-19. During the disease in patients with moderate and severe COVID-19, the hemostatic system was characterized by a procoagulant status, which persists during the period of convalescence. Fibrinolysis shutdown was detected in both moderate and severe patients with COVID-19. The procoagulant status of the coagulation system and the shutdown of fibrinolysis are typical for patients with moderate to severe COVID-19. In convalescents, activation of coagulation remains, which indicates the need to monitor the hemostatic system after Illness.

## 1. Introduction

The COVID-19 (COronaVIrus Disease 2019) pandemic, which lasted from March 2020 to May 2023, was caused by Severe Acute Respiratory Syndrome-related COronaVirus 2 (SARS-CoV-2). To the dismay of all, more than 770 million cases of the disease and almost 7 million deaths have occurred so far [1]. During this period, all the efforts worldwide were directed at investigating SARS-CoV-2, its ways of penetration into the human body, and the major pathogenetic mechanisms by which the virus can influence human beings. SARS-CoV-2 is known to be a non-segmented single-stranded RNA virus, which belongs to the *Coronaviridae* family, genus *Betacoronavirus* [2]. The virus consists of 4 structural proteins (surface glycoprotein S, envelope protein E, membrane protein M, and nucleocapsid protein N) [3], 16 nonstructural proteins (NSP 1–16) [4], and accessory proteins of open reading frames (ORF) [5]. There is supporting evidence indicating that the primary mechanism for viral penetration into host cells is through the interaction of the virus’s spike protein S with angiotensin, converting the enzyme 2 located on the surface of human cells [6]. Furthermore, this process occurs with the participation of transmembrane serine protease 2 [7] or by engaging with the membrane protein basigin [8].

The mortality risk of COVID-19 is 10–15 times higher compared to other diseases caused by human coronaviruses [9]. There is evidence of the impact of SARS-CoV-2 on all of the human organ systems [10]. For instance, it is already widely known that SARS-CoV-2 and the virus COVID-19 are able to activate the immune system and platelets and lead to dysregulation of thrombin formation as a result. It is important to emphasize that thrombin formation occurs both systemically and locally (e.g., in the lungs). All of the above leads to fibrin deposition with subsequent tissue damage and microangiopathic disorders [11,12]. At the same time, endothelium anticoagulant function decreases due to SARS-CoV-2’s penetration into the endothelial cells and the hyperactivation of the immune system and platelets; this leads to the excessive formation of thrombin and the suppression of fibrinolysis [13]. All the circumstances enumerated above are followed by the development of functional disorders of organs and organ systems [14,15]. According to the latest data, hypercoagulability is the main cause of multiple organ failure in critically ill COVID-19 patients [16].

Meanwhile, not all of the existing analyses of the coagulation system allow for an evaluation of the presence of hypercoagulation. Thus, methodological limitations of clotting methods (activated partial thromboplastin time, prothrombin time/international normalized ratio—PT/INR) do not allow us to detect hypercoagulation and microvascular thrombosis [17,18]. At the same time, viscoelastic tests, such as thromboelastography and rotational thromboelastometry (ROTEM), are more effective than coagulation tests because whole blood is sampled, providing information about the contribution of all blood cells, from factor XIII to the clot strength, its elasticity and formation speed, and fibrinolysis system activity [19].

Based on the foregoing, the aim of the study was to assess the coagulation system status and fibrinolytic activity using ROTEM in patients with COVID-19 and convalescents.

## 2. Materials and Methods

The observational prospective study included 141 patients with COVID-19:

1. Group 1—patients with mild COVID-19 (*n* = 39);
2. Group 2—patients with moderate COVID-19 (*n* = 65);
3. Group 3—patients with severe COVID-19 (*n* = 37).

The severity of COVID-19 was graded according to the interim guidelines of the Ministry of Health of the Russian Federation: “Prevention, diagnosis and treatment of a new coronavirus infection (COVID-19)” (Table 1).

The study was carried out in the following places:

1. The National Medical Research Center for Obstetrics, Gynecology and Perinatology, named after academician V.I. Kulakov of the Ministry of Health of the Russian Federation, Moscow, Russia.
2. F.I. Inozemtsev City Clinical Hospital, Moscow, Russia.
3. A database containing 18 patients with severe COVID-19 was provided by Dr. Fazoil Ataullakhanov (CTP FHF RAS, research work “Use of the thrombodynamics test in COVID-19: identification of early predictors of the development of severe pneumonia and development of effective measures for its prevention”, registration number: AAAA-A20-120111090014-6) [20].

Inclusion criteria:

- age over 18 years;
- signed informed consent.

Non-inclusion criteria:

- pregnancy or lactation;
- hereditary deficiency of blood coagulation factors predisposing to hemorrhagic conditions;
- purpura and other hemorrhagic conditions;
- cancer comorbidity;
- history of organ transplantation;
- HIV infection;
- syphilis;
- other acute infectious diseases;
- continuous use of anticoagulants/antiplatelet agents.

Exclusion criteria:

- the need for surgery during COVID-19;
- patient’s refusal to continue participation in the study.

In patients with mild COVID-19 (Group 1), the assessment was performed twice: on the 3rd–7th day of illness (point 1) and during the 14–28 days after recovery (point 2). In patients with moderate (Group 2) and severe (Group 3) COVID-19, the assessment was performed twice as well: during hospital stay (point 1); –>14 days after point 1 (point 2). No cases of acute thrombosis occurred during the study. All patients were discharged from the hospital in a satisfactory condition.

The following tests were carried out: complete blood count (CBC) was performed with automatic hematological analyzer SYSMEX XT-4000i (Sysmex Corporation, Kobe, Japan); hemostasis tests (fibrinogen according to Clauss, D-dimer) were performed with an automatic coagulometer ACL TOP 750 (Werfen, Bedford, MA, USA) using HemosIL reagents; ROTEM was performed using a thromboelastometer (ROTEM delta, Munich, Germany). The tissue factor was added as an activator in the EXTEM test (assessment of the external coagulation pathway), and ellagic acid was added in the INTEM test (assessment of the internal coagulation pathway). The FIBTEM test (assessment of the functional activity of fibrinogen) applied the tissue factor together with cytochalasin D, which prevents changes in the platelet cytoskeleton; due to this, the clot formation depended only on the functional activity of fibrinogen [21]. The parameters of ROTEM were as follows: Clotting Time (CT, s)—the time before the formation of the clot (until the clot density reaches 2 mm); Clot Formation Time (CFT, s)—the time required to increase the density of the clot from 2 to 20 mm, which characterizes the kinetics of clot formation; A5, A10, A20 (mm)—the density of the clot measured at 5, 10, 20 min after CT, Maximum Clot Firmness (MCF, mm); ML (%)—fibrinolysis, characterized by maximum lysis, which is defined as a decrease in clot density, expressed as a percentage of MCF; the calculated value of TPI (c.u.) is the thrombotic readiness index (TPI = EMX/K, where EMX = (100 × MCF)/(100 − MCF)).

S-Monovette^®^ tubes (Sarstedt, Germany) were used for blood sampling from the cubital vein following an overnight fast. Tubes with anticoagulant sodium citrate at a concentration of 109 mM (in a ratio of 1:9) were used to analyze hemostasis. Centrifugation for 20 min (1500× *g*) using an SM-6 centrifuge (ELMI, Riga, Latvia) was performed to obtain platelet-poor plasma (PPP).

Statistical analysis was performed using Microsoft Excel (Excel 2013, Microsoft, Redmond, WA, USA), MedCalc® Software (version 20.113, MedCalc Software Ltd., Ostend, Belgium) and the Statistica V10 statistical software package (version 10.0.1011.0, Statistica, New York, NY, USA). Risks (%) were calculated to evaluate qualitative data. The χ^2^ test was used to compare categorical data in two or more groups. The type of quantitative data distribution was determined by the Kolmogorov–Smirnov test and visual data analysis. Mean values with a standard deviation (M ± SD) were calculated for the data with a normal distribution, and parametric statistics were used (ANOVA in 3 groups and pared *t*-test in 2 groups). For nonparametric data, median values (Me) with an interquartile range (Q1–Q3) were calculated, and the nonparametric statistics were used (Kruskal–Wallis test in 3 groups and matched-pairs Wilcoxon signed-rank test in 2 groups). The correlation analysis was performed using the Spearman’s rank correlation test. Differences were considered statistically significant at *p* < 0.05.

Ethical approval has been obtained from the local ethics committee of the National Medical Research Center for Obstetrics, Gynecology and Perinatology, named after academician V.I. Kulakov of the Ministry of Health of the Russian Federation.

## 3. Results

We studied the clinical and laboratory data of patients in three groups depending on the COVID-19 severity (Table 2). Patients with mild and moderate COVID-19 were mostly women (32 (82.1%) and 41 (63.1%), respectively), and patients with severe COVID-19 were mostly men—22 (59.5%) (*p* = 0.0009). Patients with moderate and severe COVID-19 were older (Me = 60 (Q1–Q3 = 43–78) and Me = 63 (Q1–Q3 = 53–71) years) than the mild COVID-19 patients (Me = 38 (Q1–Q3 = 34–54) years (*p* < 0.0001)). The threshold age determining the severity of the disease was 56 years: there were 8 (20.5%), 41 (63.1%), and 25 (67.6%) patients over 56 in the groups with mild, moderate, and severe COVID-19, respectively, *p* < 0.0001. An increase in body mass index (BMI) correlated significantly with the severity of the infection: patients overweight and with obesity (BMI ≥ 25 kg/m^2^) prevailed in the group with severe COVID-19 (*p* = 0.0138). At the same time, the mean BMI was excessive in all groups of patients: 25.2 ± 4.4 (Group 1), 27.4 ± 6.2 (Group 2), and 29.1 ± 5.3 (Group 3); *p* = 0.0092. Patients’ height did not differ among the groups.

According to ROTEM thromboelastometry, the clotting time of CT EXTEM was prolonged in Groups 2 and 3 at point 1. The clot formation time (CFT EXTEM) in Group 3 tended to be shorter at both points (Table 3).

The CT INTEM clotting time did not differ in the groups and remained within the reference intervals (RI) at both points of the study. A shortening of CFT INTEM at time point 1 was observed in Groups 2 and 3, but at point 2, it was shortened only in the convalescents of Group 3 (Table 4).

The EXTEM α-angle and the INTEM α-angle were larger in patients with severe COVID-19 at point 1, and in Group 1 these parameters tended to decrease at point 2. According to the EXTEM test (A10, A20, MCF), an increase in clot density was noted at point 1 in Groups 2 and 3, but at point 2, only Group 3 remained the same. The clot density estimated by the INTEM test was similar to the EXTEM test. The A10 and A20 FIBTEM test parameters at point 1 exceeded the RI in Groups 2 and 3, whereas the maximum clot density (MCF) exceeded the RI only in Group 3 (Table 5).

**Figure 1:**
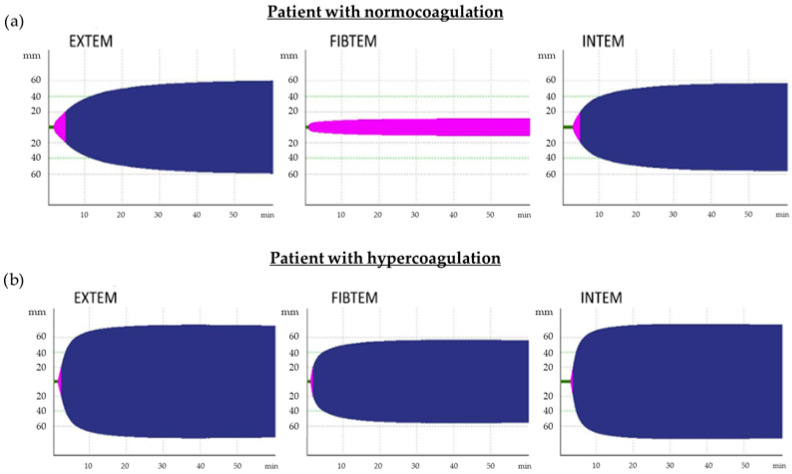
TEMograms, which are typical for patients with normocoagulation (**a**) and hypercoagulation (**b**). EXTEM and INTEM TEMograms of patients with hypercoagulation are characterized by a reduction in time before the formation of the clot (Clotting Time (CT), green color) and the time from CT until clot density reaches 20 mm (Clot Formation Time (CFT), pink color) as well as by an increase in clot density (indicated by the amplitude of the maximum clot firmness (MCF), blue color) because of an increase in fibrinogen levels and activation of platelets. For patients with normocoagulation (indicated by the amplitude of MCF on FIBTEM TEMogram, which does not exceed 20 mm, pink color), formation of clots only due to fibrin is typical (platelets are blocked by cytocholosin), but in patients with hypercoagulation, clots are formed both due to fibrin and platelets; therefore, the MCF FIBTEM increases and exceeds 20 mm (blue color).

According to the results of the intergroup comparison, the thrombodynamic potential index (TPI EXTEM, INTEM) reflected the dependence of the degree of hypercoagulation on the severity of COVID-19 both during illness and during convalescence. All patients with COVID-19 are characterized by TEMograms (FIBTEM, EXTEM, INTEM) with increased clot density, reflecting severe hypercoagulation (Figure 1).

**Figure 2:**
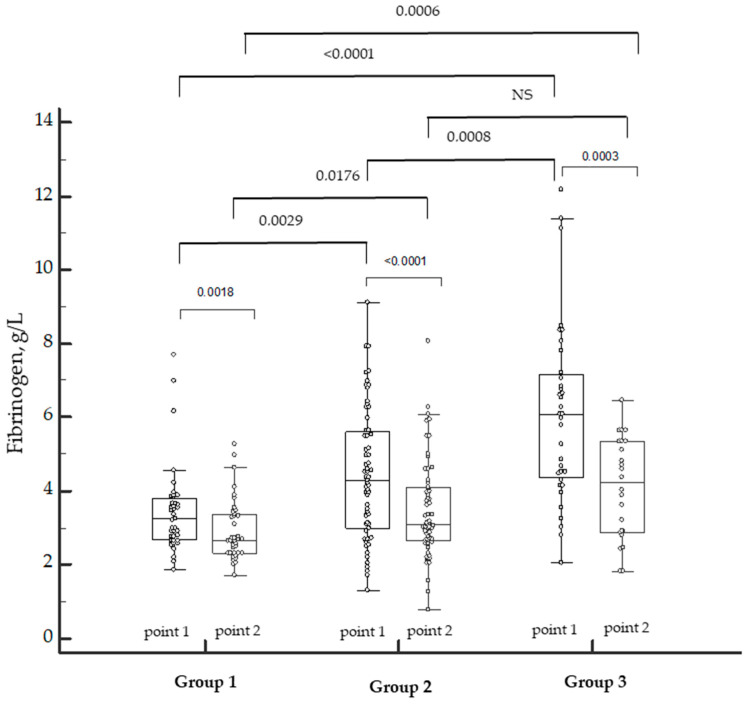
Dynamics of fibrinogen levels in patients with COVID-19 of varying severity during the disease and in the convalescence period. NS—not significant. Individual patient data marked by the white dots. *p*-values marked above the bar graphs.

Since the density of the clot depends on the level of fibrinogen, we incorporated it into the dynamics between the groups included in the investigation (Figure 2).

The levels of fibrinogen depended on the severity of COVID-19. The levels were higher in Group 3 during the disease (point 1) as well as in the convalescence period (point 2). The positive dynamics of a decrease in the levels of fibrinogen were observed in all the groups. However, in our study, the excess of fibrinogen remained in the convalescents of Group 3, and three patients of Group 2 were diagnosed with hypofibrinogenemia.

We also found that, in patients with COVID-19 (point 1), clot density correlated with the fibrinogen level (r = 0.5263; *p* < 0.001) and platelet count (r = 0.4435; *p* < 0.001).

Moreover, the dependence of MCF EXTEM on the fibrinogen level, the number of platelets, and neutrophils was determined in patients with different severity levels of COVID-19. Group 1 showed correlations between MCF EXTEM and fibrinogen levels (r = 0.5222; *p* < 0.05), MCF EXTEM and platelets (0.2408; *p* < 0.05), and MCF EXTEM and neutrophils (−0.1527; *p* < 0.05). This finding confirms that the main contribution to clot strength is made by fibrinogen but not by blood cells. In Group 2, an increased contribution of platelets and neutrophils to the clot strength was noted: r = 0.5193 (*p* < 0.05), r = 0.4882 (*p* < 0.05), respectively. Group 3 showed correlations between MCF EXTEM and fibrinogen (r = 0.6179; *p* < 0.001) and between MCF EXTEM and platelets (r = 0.7513; *p* < 0.001) but no correlation with neutrophils.

In patients with more a severe course of COVID-19, the number of neutrophils was higher; there was also a tendency for the platelets to increase (Table 6), but the clot density primarily depended on fibrinogen and platelets.

**Figure 3:**
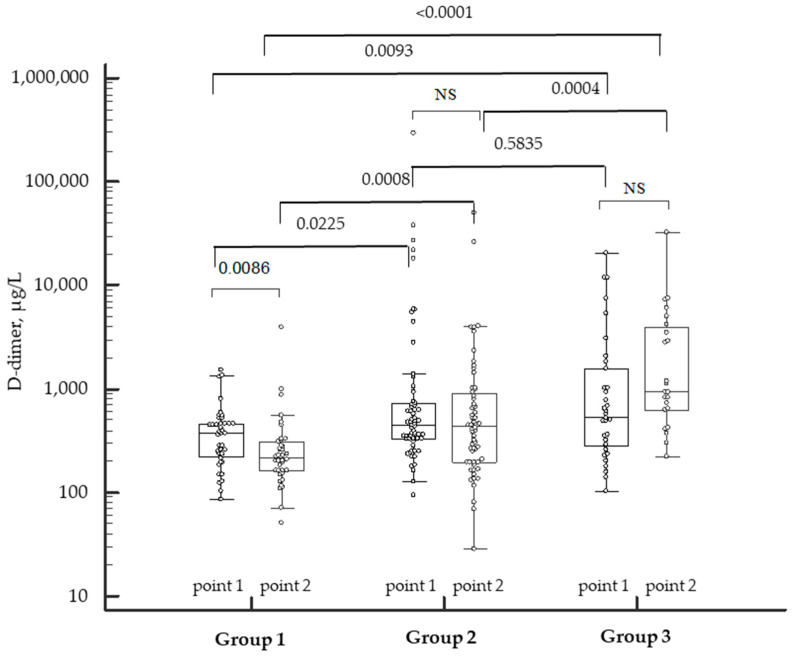
Dynamics of D-dimer levels in patients with COVID-19 of varying severity during the disease and in the convalescence period. NS—not significant. Individual patient data marked by the white dots. *p*-values marked above the bar graphs.

COVID-19-related hypercoagulation led to the activation of fibrinolysis, of which a laboratory marker was D-dimer. The levels of D-dimer were higher in patients with moderate and severe COVID-19. A decrease in the levels of D-dimer relative to the baseline was noted in patients with mild COVID-19 during the convalescence period. At the same time, an elevation in this marker was found in patients with severe COVID-19 despite the therapy, which indicated continued activation of the coagulation (Figure 3). Surprisingly, a wide range of the levels of D-dimer within the same group was observed.

The activity of the fibrinolysis system (ML EXTEM, ML INTEM) during COVID-19 did not differ between the groups, but it was reduced in convalescents after moderate and severe COVID-19. The disabling of fibrinolysis by ROTEM is stated at ML EXTEM < 3.5% in a 60 min trial [19,21].

**Figure 4:**
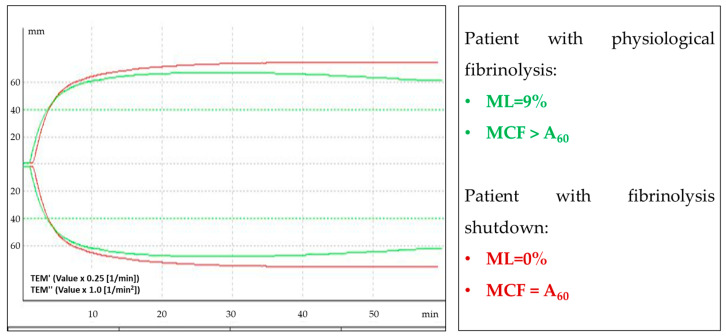
Example of an EXTEM TEMogram of patients with physiological fibrinolysis (green curve) and TEMogram of patients with fibrinolysis shutdown (red curve). The density of a clot normally decreases up to 15% for 60 min after reaching the maximum clot firmness (MCF) (this parameter is indicated by ML (fibrinolysis, characterized by maximum lysis)). In patients with fibrinolysis shutdown, the clot density does not decrease (ML = 0%).

In our study, decreased clot lysis (ML EXTEM < 3.5%) was found in eight patients: in six (75%) with moderate COVID-19 and in two (25%) with severe COVID-19. TEMograms that are typical for patients with physiological fibrinolysis and fibrinolysis shutdown are shown in Figure 4.

According to FIBTEM, fibrinolytic activity increased in patients of Groups 2 and 3 at point 1 but did not differ in the convalescence period (point 2).

## 4. Discussion

Our study showed that male gender, older age, overweight, and obesity are risk factors for severe COVID-19; this finding is consistent with the data from other studies [22,23,24]. ACE2 expression in alveolar epithelial cells is higher in men than in women; this may contribute to a more severe course of COVID-19 in men [25]. There are convincing data that obesity and being overweight increase the chance of developing severe forms of COVID-19. This is associated with a combination of obesity, somatic and endocrine diseases, and metabolic and immune disorders [26].

CT EXTEM in patients with moderate and severe COVID-19 revealed the deficiency of external coagulation pathway factors. Along with this, the chronometric parameters CFT EXTEM, CFT INTEM, and the EXTEM/INTEM α-angle indicated hypercoagulation in patients with moderate and severe COVID-19. An increased α-angle showed a significant role of platelets in the coagulation hyperactivation, which is caused by the thrombin release and the blood coagulation cascade. The structural parameters of the tests (A10, A20, MCF), characterizing the size and density of the clot, made up the degrees of hypercoagulation in patients with COVID-19 of varying severity, consistent with previously obtained data [27]. Several studies prior to the COVID-19 pandemic showed that MCF > 68 mm or A10 > 61.5 mm by EXTEM or INTEM tests indicate hypercoagulation and are predictors of thromboembolic complications [25]. In our study, Group 3 patients had a high risk of thromboembolic complications. It is important to note that in this group, the risk of thrombosis remains for the period of convalescence; this was confirmed by the test results at point 2. MCF FIBTEM > 25 mm is another criterion for a high risk of thromboembolic complications [26] in patients with moderate and severe on-going COVID-19 and in convalescents after severe disease; this also indicates persistent hypercoagulation and the need to monitor hemostasis after recovery.

Previous studies have shown that, in trauma and cancer patients, maximum clot density MCF EXTEM correlates with fibrinogen and platelet levels [19]. We have also shown the dependence of clot density (MCF) on fibrinogen levels and platelet and neutrophil counts in patients with COVID-19. It is believed that the expression of SARS-CoV-2 proteins ORF3a, NSP1, and NSP13 activates the nod-like receptor protein 3 (NLRP3) inflammatory pathway [28,29], which is known to play a central role in initiating the inflammatory cascade in several diseases [30]. Clinical studies show the crucial role of the NLPR3 inflammasome in the development of moderate or severe COVID-19 [31]. Thus, the release of extracellular neutrophil traps in conjunction with NLRP3 inflammasome activation promotes the development of an uncontrolled pathological thromboinflammation through platelet activation, augmentation and densification of fibrin clots, epithelium damage, and violation of the integrity of blood vessels [32], finally leading to ischemia [33].

Since thrombin is formed in excess during COVID-19, the fibrinolysis system is activated to prevent the occlusion of vessels. But there are cases when this mechanism of regulation is disrupted, which can lead to shutdown fibrinolysis [34]. Distinctive features of COVID-19 are endothelial damage, inflammation, and activation of the coagulation cascade associated with the severity of COVID-19, leading to a stable prothrombotic state of hemostasis [35], which is exacerbated by frequent fibrinolysis shutdown [21]. Madathil et al. reported that, in patients with severe COVID-19, a fibrinolysis shutdown (ML EXTEM, ML INTEM = 0%) correlated with thromboembolic complications and renal failure, despite high levels of D-dimer [36]. We have noted a wide range of levels of D-dimer within the same group in our study. This fact might be due to the individual characteristics of the endothelium state prior to COVID-19 and its resistance to damage [37]. Meanwhile, a combination of D-dimer levels (>2.6 μg/mL) and fibrinolysis shutdown (ML = 0%) had the best predictive value for thromboembolic complications: the rate of thrombotic events under this association was 50%, and the rate of renal failure with the need for dialysis was 80% [38]. In our study, four patients with moderate to severe COVID-19, reduced fibrinolysis, and high D-dimer levels were included in the high-risk group for thromboembolic complications and multiple organ failure. There is evidence of hypercoagulation [39], vasculopathy [40,41], and cardiovascular disorders in patients with COVID-19 [42].

## 5. Conclusions

In patients with moderate and severe COVID-19, a procoagulant status of hemostasis manifested with high levels of thrombinemia and platelet activation; an increase in the functional activity of fibrinogen; and an increase in clot density due to blood cells. The suppression of fibrinolytic activity with the simultaneous activation of the coagulation system, detected by ROTEM, can be considered as a predictor of thromboembolic complications in COVID-19 patients. In patients with severe COVID-19, the procoagulant status of the hemostasis persists after recovery, which indicates the need for laboratory monitoring during convalescence.

## Figures and Tables

**Table 1:** Criteria for the severity of COVID-19.

Criteria	Mild COVID-19	Moderate COVID-19	Severe COVID-19
Complaints and main symptoms	Cough Weakness Sore throat	Shortness of breath during physical activity	Altered level of consciousness, agitation
Body temperature, °C	<38	>38	-
Respiratory rate, bits per min	-	>22	>30
Saturation (SpO_2_), %	-	<95	≤93
CT/X-ray data	-	Typical for viral infection	-
serum CRP, mg/L	-	>10	-
PaO_2_/FiO_2_, mmHg	-	-	≤300
Arterial blood lactate, mmol/L	-	-	>2
Hemodynamics	-	-	Unstable (systolic blood pressure less than 90 mm Hg or diastolic blood pressure less than 60 mm Hg, diuresis less than 20 mL/h)
qSOFA, points	-	-	>2

qSOFA—quick Sepsis-Related Organ Failure Assessment.

**Table 2:** Clinical characteristics of patients.

Characteristics	Group 1	Group 2	Group 3	*p*-Value
Gender male	7 (17.9%)	24 (36.9%)	22 (59.5%)	0.0009 ***
Gender female	32 (82.1%)	41 (63.1%)	15 (40.5%)	
Age, years	38 (34–54)	60 (43–78)	63 (53–71)	0.0001 **
Age ≥ 56 years	8 (20.5%)	41 (63.1%)	25 (67.6%)	<0.0001 ***
Height, m	1.67 ± 0.09	1.68 ± 0.08	1.69 ± 0.07	0.5664 *
Body weight, kg	71.3 ± 15.4	78.5 ± 20.3	83.3 ± 12.5	0.0094 *
BMI, kg/m^2^	25.2 ± 4.4	27.4 ± 6.2	29.1 ± 5.3	0.0092 *
Patients with BMI ≥ 25 kg/m^2^	19 (48.7%)	34 (52.3%)	29 (78.4%)	0.0138 ***
Smoking	4 (10.3%)	10 (15.4%)	1 (2.7%)	0.1355 ***
Blood group 0(I)	18 (46.1%)	9 (13.9%)	10 (27.0%)	0.0017 ***
Blood group A(II)	12 (30.8%)	42 (64.6%)	18 (48.7%)	
Blood group B(III)	3 (7.7%)	11 (16.9%)	7 (18.9%)	
Blood group AB(IV)	6 (15.4%)	3 (4.6%)	2 (5.4%)	
Alcohol consumption	13 (33.3%)	12 (18.5%)	9 (24.3%)	0.229 ***
Duration of treatment, days	7.4 ± 5.1	12.1 ± 10.4	30.0 ± 7.4	0.0026 ***

* M ± Sd, ANOVA; ** Me (Q1–Q3), Kruskal–Wallis test; *** *n* (%), ꭓ^2^-test.

**Table 3:** EXTEM parameters in COVID-19 patients and convalescents.

Indicator, Measurement Units, (Reference Interval)	Time Point	Group 1	Group 2	Group 3	*p*-Value
CT EXTEM, s, (38–79)	1	67 (62.3–72.8)	79.9 (67–106)	81 (70.5–106.8)	<0.0001 *
	2	72.5 (70–98)	79 (67–106)	77 (74.3–85.8)	0.7174 *
		*p* < 0.0001 **	*p* = 0.6608 **	*p* = 0.2946 **	
CFT EXTEM, s, (34–159)	1	77 (67.5–91.8)	70 (62–86.5)	57 (50.5–72)	0.0043 *
	2	80 (65–95)	68.5 (57–88)	58 (44–64.5)	0.0010 *
		*p* = 0.9531 **	*p* = 0.8044 **	*p* = 0.3328 **	
α-angle EXTEM, %, (64–79)	1	75 (72–77)	76 (73–78)	78 (75.8–80)	0.0043 *
	2	74 (71–77)	76.5 (73–79)	78 (78–81.5)	0.0006 *
		*p* = 0.7566 **	*p* = 0.7809 **	*p* = 0.4855 **	
A10 EXTEM, mm, (43–65)	1	59 (53.3–60.8)	60.5 (56–66)	64 (58.5–70.5)	0.0074 *
	2	58.5 (53–64)	60 (55–66)	64 (58.5–70.5)	0.0054 *
		*p* = 0.9491 **	*p* = 0.8167 **	*p* = 0.5141 **	
A20 EXTEM, mm, (55–72)	1	64 (59.3–66)	66 (62–71)	70 (62.8–73.3)	0.0131 *
	2	64 (60–68)	66 (62–71)	69 (65–74.8)	0.0065 *
		*p* = 0.7683 **	*p* = 0.9249 **	*p* = 0.5398 **	
MCF EXTEM, mm, (50–72)	1	65 (60–67)	66 (62–72)	70 (66.3–73.3)	0.0162 *
	2	64.5 (60–69)	67.5 (64–71)	70 (66.3–74.7)	0.0031 *
		*p* = 0.8328 **	*p* = 0.5319 **	*p* = 0.4883 **	
TPI EXTEM, c.u., (19–131)	1	74.5 (52–86)	87 (59.8–135)	126 (75–154)	0.0084 *
	2	71.5 (46–104)	86 (61.5–135.3)	122 (92–182.8)	0.0007 *
		*p* = 0.9204 **	*p* = 0.7217 **	*p* = 0.4291 **	
ML EXTEM, %, <15	1	6 (3.3–9)	6 (4–11)	7 (2.8–9.3)	0.8242 *
	2	5 (3–9)	4 (2–6)	3 (2–4)	0.0256 *
		*p* = 0.4852 **	*p* = 0.0004 **	*p* = 0.0484 **	

EXTEM—assessment of the external coagulation pathway; CT—clotting time; CFT—clot formation time; A10, A20—density of the clot measured at 5, 10, 20 min after CT; MCF—maximum clot firmness; TPI—thrombotic readiness index; ML—fibrinolysis, characterized by maximum lysis. Note: reference intervals (RI) were calculated in the laboratory of the National Medical Research Center for Obstetrics, Gynecology and Perinatology, named after academician V.I. Kulakov of the Ministry of Health of the Russian Federation; * Me (Q1–Q3), Kruskal–Wallis test; ** matched-pairs Wilcoxon signed-rank test.

**Table 4:** INTEM parameters in COVID-19 patients and convalescents.

Indicator, Measurement Units, (Reference Interval)	Time Point	Group 1	Group 2	Group 3	*p*-Value
CT INTEM, s, (100–240)	1	194 (178–212)	190 (174–206)	198 (189–224)	0.1924 *
	2	200 (192–214)	193 (172–209)	201 (181–216)	0.1459 *
		*p* = 0.1453 **	*p* = 0.7569 **	*p* = 0.4408 **	
CFT INTEM, s, (30–110)	1	70 (62–90)	61.5 (49–72)	56 (45.3–64.3)	0.0002 *
	2	80 (65–95)	68.5 (57–88)	58 (44–64.5)	0.0010 *
		*p* = 0.3219 **	*p* = 0.7630 **	*p* = 0.2511 **	
α-angle INTEM, %, (64–80)	1	76 (73–77)	77 (75–80)	79 (76.8–81)	0.0043 *
	2	74 (71–77)	76.5 (73–79)	78 (78–81.5)	0.0006 *
		*p* = 0.7302 **	*p* = 0.9332 **	*p* = 0.1761 **	
A10 INTEM, mm, (44–66)	1	56 (52–60)	60 (56–64.5)	63 (57.5–68.5)	0.0021 *
	2	58.5 (53–64)	60 (55–66)	64 (58.5–70.5)	0.0054 *
		*p* = 0.6201 **	*p* = 0.8084 **	*p* = 0.4641 **	
A20 INTEM, mm, (55–70)	1	61 (57.3–64)	64 (61–68.5)	65 (62.3–73.3)	0.0029 *
	2	64 (60–68)	66 (62–71)	69 (65–74.8)	0.0065 *
		*p* = 0.3929 **	*p* = 0.3915 **	*p* = 0.3951 **	
MCF INTEM, mm, (50–71)	1	60 (58–64.8)	64 (61–69)	65 (63.7–71.5)	0.0023 *
	2	64.5 (60–69)	67.5 (64–71)	70 (66.3–74.8)	0.0031 *
		*p* = 0.4856 **	*p* = 0.2896 **	*p* = 0.3227 **	
TPI INTEM, c.u., (39–143)	1	65.5 (47–85)	87 (63.5–136)	128.5 (80–172.5)	0.0005 *
	2	73 (52.3–97.5)	93 (73.5–135.5)	126.5 (102–126.5)	<0.0001 *
		*p* = 0.4386 **	*p* = 0.4443 **	*p* = 0.3947 **	
ML INTEM, %, <15	1	7 (5–10)	7 (4.5–12)	7 (1.8–11.3)	0.8655 *
	2	6 (4–8.8)	4.5 (2–8)	3 (0–4.8)	0.0235 *
		*p* = 0.3325 **	*p* = 0.0013 **	*p* = 0.0290 **	

INTEM—assessment of the internal coagulation pathway; CT—clotting time; CFT—clot formation time; A10, A20—density of the clot measured at 5, 10, 20 min after CT; MCF—maximum clot firmness; TPI—thrombotic readiness index; ML—fibrinolysis, characterized by maximum lysis. Note: reference intervals (RI) were calculated in the laboratory of the National Medical Research Center for Obstetrics, Gynecology and Perinatology, named after academician V.I. Kulakov of the Ministry of Health of the Russian Federation; * Me (Q1–Q3), Kruskal–Wallis test; ** matched-pairs Wilcoxon signed-rank test.

**Table 5:** FIBTEM parameters in COVID-19 patients and convalescents.

Indicator, Measurement Units, (Reference Interval)	Time Point	Group 1	Group 2	Group 3	*p*-Value
A 10 FIBTEM, mm, (8–23)	1	17 (14.3–22)	23.5 (17–33)	28 (19–35.3)	0.0004 *
	2	17 (13–20)	21 (17–27)	27 (22.3–34.3)	<0.0001 *
		*p* = 0.2869 **	*p* = 0.1647 **	*p* = 0.9056 **	
A 20 FIBTEM, mm, (9–25)	1	18 (15–23)	25.5 (18–35)	29 (20–37.3)	0.0002 *
	2	17 (14–21)	23 (19–30)	28 (23.5–35.8)	<0.0001 *
		*p* = 0.3048 **	*p* = 0.1739 **	*p* = 0.8744 **	
MCF FIBTEM, mm, (10–28)	1	18 (15.3–23.7)	26 (18.5–35.5)	29 (20–37.5)	0.0001 *
	2	17 (14–21)	24 (18–30)	28.1 (24.5–36)	<0.0001 *
		*p* = 0.2865 **	*p* = 0.1337 **	*p* = 0.8588 **	
ML FIBTEM, %, <9	1	0 (0–1)	1 (0–4.5)	1 (0–3.3)	0.0232 *
	2	0 (0–1)	0 (0–3)	0 (0–2.3)	0.3781 *
		*p* = 0.7827 **	*p* = 0.2770 **	*p* = 0.1015 **	

FIBTEM—assessment of the functional activity of fibrinogen; A10, A20—density of the clot measured at 5, 10, 20 min after clotting time; MCF—maximum clot firmness; ML—fibrinolysis, characterized by maximum lysis. Note: reference intervals (RI) were calculated in the laboratory of the National Medical Research Center for Obstetrics, Gynecology and Perinatology, named after academician V.I. Kulakov of the Ministry of Health of the Russian Federation; * Me (Q1–Q3), Kruskal–Wallis test; ** matched-pairs Wilcoxon signed-rank test.

**Table 6:** Parameters of a complete blood count in patients with on-going COVID-19 of varying severity.

Indicator (Measurement Units) (Reference Interval)	Group 1	Group 2	Group 3	*p*-Value
Leukocytes (×10^9^/L) (3.50–9.00)	5.12 ± 2.29	6.16 ± 3.41	7.48 ± 3.21	0.0060 *
Red blood cells (×10^12^/L) (3.50–5.10)	4.86 ± 0.49	4.50 ± 0.59	4.67 ± 0.73	0.0197 *
Hemoglobin (g/L) (105–170)	139.9 ± 15.7	132.1 ± 24.1	132.1 ± 24.5	0.1893 *
Hematocrit (0.320–0.460)	0.417 ± 0.049	0.390 ± 0.064	0.386 ± 0.064	0.0451 *
Platelets (×10^9^/L) (150–400)	239.8 ± 52.2	235.7 ± 90.3	260.5 ± 110.1	0.3871 *
Neutrophils (×10^9^/L) (2.0–6.0)	2.97 ± 2.11	4.02 ± 2.43	5.85 ± 2.99	<0.0001 *

* M ± Sd, ANOVA.

## Data Availability

The data presented in this study are available on request from the corresponding author. The data are not publicly available due to privacy or ethical restrictions.

## References

[1] WHO (2023). WHO Coronavirus (COVID-19) Dashboard.

[2] Lu R., Zhao X., Li J., Niu P., Yang B., Wu H., Wang W., Song H., Huang B., Zhu N. (2020). Genomic characterisation and epidemiology of 2019 novel coronavirus: Implications for virus origins and receptor binding. Lancet.

[3] McCloskey B., Heymann D.L. (2020). SARS to novel coronavirus—Old lessons and new lessons. Epidemiol. Infect..

[4] Thomas S. (2021). Mapping the Nonstructural Transmembrane Proteins of Severe Acute Respiratory Syndrome Coronavirus 2. J. Comput. Biol..

[5] Michel C.J., Mayer C., Poch O., Thompson J.D. (2020). Characterization of accessory genes in coronavirus genomes. Virol. J..

[6] Shang J., Wan Y., Luo C., Ye G., Geng Q., Auerbach A., Li F. (2020). Cell entry mechanisms of SARS-CoV-2. Proc. Natl. Acad. Sci. USA.

[7] Zang R., Gomez Castro M.F., McCune B.T., Zeng Q., Rothlauf P.W., Sonnek N.M., Liu Z., Brulois K.F., Wang X., Greenberg H.B. (2020). TMPRSS2 and TMPRSS4 promote SARS-CoV-2 infection of human small intestinal enterocytes. Sci. Immunol..

[8] Henarejos-Castillo I., Sebastian-Leon P., Devesa-Peiro A., Pellicer A., Diaz-Gimeno P. (2020). SARS-CoV-2 infection risk assessment in the endometrium: Viral infection-related gene expression across the menstrual cycle. Fertil. Steril..

[9] Chan J.F.-W., Yuan S., Kok K.-H., To K.K.-W., Chu H., Yang J., Xing F., Liu J., Yip C.C., Poon R.W. (2020). A familial cluster of pneumonia associated with the 2019 novel coronavirus indicating person-to-person transmission: A study of a family cluster. Lancet.

[10] Huang C., Wang Y., Li X., Ren L., Zhao J., Hu Y., Zhang L., Fan G., Xu J., Gu X. (2020). Clinical features of patients infected with 2019 novel coronavirus in Wuhan, China. Lancet.

[11] Chowdary P. (2022). COVID-19 coagulopathy—What should we treat?. Exp. Physiol..

[12] Beznoshenko O., Gorodnova E., Dolgushina N., Krechetova L., Ivanets T., Romanov A., Menzhinskaya I.V., Piregov A.V. (2022). Generation of the Thrombin in COVID-19 Patients with Thrombophilia of Various Genesis Both during Acute Phase and Recovery. Int. J. Bioeng. Life Sci..

[13] Pavoni V., Gianesello L., Pazzi M., Dattolo P., Prisco D. (2022). Questions about COVID-19 associated coagulopathy: Possible answers from the viscoelastic tests. J. Clin. Monit. Comput..

[14] Kuri-Cervantes L., Pampena M.B., Meng W., Rosenfeld A.M., Ittner C.A.G., Weisman A.R., Agyekum R.S., Mathew D., Baxter A.E., Vella L.A. (2020). Comprehensive mapping of immune perturbations associated with severe COVID-19. Sci. Immunol..

[15] Lucas C., Wong P., Klein J., Castro T.B.R., Silva J., Sundaram M., Ellingson M.K., Mao T., Oh J.E., Israelow B. (2020). Longitudinal analyses reveal immunological misfiring in severe COVID-19. Nature.

[16] Pavoni V., Gianesello L., Pazzi M., Stera C., Meconi T., Frigieri F.C. (2020). Evaluation of coagulation function by rotation thromboelastometry in critically ill patients with severe COVID-19 pneumonia. J. Thromb. Thrombolysis.

[17] Lodigiani C., Iapichino G., Carenzo L., Cecconi M., Ferrazzi P., Sebastian T., Kucher N., Studt J.-D., Sacco C., Bertuzzi A. (2020). Venous and arterial thromboembolic complications in COVID-19 patients admitted to an academic hospital in Milan, Italy. Thromb. Res..

[18] Yao Y., Cao J., Wang Q., Shi Q., Liu K., Luo Z., Chen X., Chen S., Yu K., Huang Z. (2020). D-dimer as a biomarker for disease severity and mortality in COVID-19 patients: A case control study. J. Intensive Care.

[19] Bareille M., Hardy M., Douxfils J., Roullet S., Lasne D., Levy J.H., Stépanian A., Susen S., Frère C., Lecompte T. (2021). Viscoelastometric Testing to Assess Hemostasis of COVID-19: A Systematic Review. J. Clin. Med..

[20] Krechetova L.V., Nechipurenko D.Y., Shpilyuk M.A., Beznoshchenko O.S., Beresneva E.A., Markelov M.I., Ivanets T.Y., Gavrilova T.Y., Kozachenko I.F., Esayan R.M. (2021). The use of the thrombodynamics test in the diagnostics of hemostasis disorders in patients with COVID-19 of varying severity. J. Clin. Pract..

[21] Mitrovic M., Sabljic N., Cvetkovic Z., Pantic N., Dakic A.Z., Bukumiric Z., Libek V., Savic N., Milenkovic B., Virijevic M. (2021). Rotational thromboelastometry (ROTEM) profiling of COVID–19 patients. Platelets.

[22] Chen N., Zhou M., Dong X., Qu J., Gong F., Han Y., Qiu Y., Wang J., Liu Y., Wei Y. (2020). Epidemiological and clinical characteristics of 99 cases of 2019 novel coronavirus pneumonia in Wuhan, China: A descriptive study. Lancet.

[23] Zhou F., Yu T., Du R., Fan G., Liu Y., Liu Z., Xiang J., Wang Y., Song B., Gu X. (2020). Clinical course and risk factors for mortality of adult inpatients with COVID-19 in Wuhan, China: A retrospective cohort study. Lancet.

[24] Henderson L.A., Canna S.W., Schulert G.S., Volpi S., Lee P.Y., Kernan K.F., Caricchio R., Mahmud S., Hazen M.M., Halyabar O. (2020). On the Alert for Cytokine Storm: Immunopathology in COVID-19. Arthritis Rheumatol..

[25] Hincker A., Feit J., Sladen R.N., Wagener G. (2014). Rotational thromboelastometry predicts thromboembolic complications after major non-cardiac surgery. Crit. Care.

[26] Zanetto A., Senzolo M., Vitale A., Cillo U., Radu C., Sartorello F., Spiezia L., Campello E., Rodriguez-Castro K., Ferrarese A. (2017). Thromboelastometry hypercoagulable profiles and portal vein thrombosis in cirrhotic patients with hepatocellular carcinoma. Dig. Liver Dis..

[27] Kong R., Hutchinson N., Görlinger K. (2021). Hyper- and hypocoagulability in COVID-19 as assessed by thromboelastometry -two case reports-. Korean J. Anesthesiol..

[28] Zhang J., Ejikemeuwa A., Gerzanich V., Nasr M., Tang Q., Simard J.M., Zhao R.Y. (2022). Understanding the Role of SARS-CoV-2 ORF3a in Viral Pathogenesis and COVID-19. Front. Microbiol..

[29] Kim N.-E., Kim D.-K., Song Y.-J. (2021). SARS-CoV-2 Nonstructural Proteins 1 and 13 Suppress Caspase-1 and the NLRP3 Inflammasome Activation. Microorganisms.

[30] Wang Z., Zhang S., Xiao Y., Zhang W., Wu S., Qin T., Yue Y., Qian W., Li L. (2020). NLRP3 Inflammasome and Inflammatory Diseases. Oxidative Med. Cell. Longev..

[31] Zhang J., Ma X., Liu F., Zhang D., Ling J., Zhu Z., Chen Y., Yang P., Yang Y., Liu X. (2023). Role of NLRP3 inflammasome in diabetes and COVID-19 role of NLRP3 inflammasome in the pathogenesis and treatment of COVID-19 and diabetes NLRP3 inflammasome in diabetes and COVID-19 intervention. Front. Immunol..

[32] Makatsariya A., Slukhanchuk E., Bitsadze V., Khizroeva J., Tretyakova M., Tsibizova V., Dobryakov A., Elalamy I., Gris J.C. (2020). COVID-19, neutrophil extracellular traps and vascular complications in obstetric practice. JPME.

[33] Singh P., Kumar N., Singh M., Kaur M., Singh G., Narang A., Kanwal A., Sharma K., Singh B., Napoli M.D. (2023). Neutrophil Extracellular Traps and NLRP3 Inflammasome: A Disturbing Duo in Atherosclerosis, Inflammation and Atherothrombosis. Vaccines.

[34] Nougier C., Benoit R., Simon M., Desmurs-Clavel H., Marcotte G., Argaud L., David J.S., Bonnet A., Negrier C., Dargaud Y. (2020). Hypofibrinolytic state and high thrombin generation may play a major role in SARS-CoV2 associated thrombosis. J. Thromb. Haemost..

[35] Dolgushina N., Gorodnova E., Beznoshenco O., Romanov A., Menzhinskaya I., Krechetova L., Sukhikh G. (2022). Von Willebrand Factor and ADAMTS-13 Are Associated with the Severity of COVID-19 Disease. J. Clin. Med..

[36] Wright F.L., Vogler T.O., Moore E.E., Moore H.B., Wohlauer M.V., Urban S., Nydam T.L., Moore P.K., McIntyre R.C. (2020). Fibrinolysis Shutdown Correlation with Thromboembolic Events in Severe COVID-19 Infection. J. Am. Coll. Surg..

[37] Xu S.-W., Ilyas I., Weng J.-P. (2022). Endothelial dysfunction in COVID-19: An overview of evidence, biomarkers, mechanisms and potential therapies. Acta Pharmacol. Sin..

[38] Görlinger K., Almutawah H., Almutawaa F., Alwabari M., Alsultan Z., Almajed J., Alwabari M., Alsultan M., Shahwar D., Yassen K.A. (2021). The role of rotational thromboelastometry during the COVID-19 pandemic: A narrative review. Korean J. Anesthesiol..

[39] Oxley T.J., Mocco J., Majidi S., Kellner C.P., Shoirah H., Singh I.P., De Leacy R.A., Shigematsu T., Ladner T.R., Yaeger K.A. (2020). Large-Vessel Stroke as a Presenting Feature of COVID-19 in the Young. N. Engl. J. Med..

[40] Magro C., Mulvey J.J., Berlin D., Nuovo G., Salvatore S., Harp J., Baxter-Stoltzfus A., Laurence J. (2020). Complement associated microvascular injury and thrombosis in the pathogenesis of severe COVID-19 infection: A report of five cases. Transl. Res..

[41] Teuwen L.-A., Geldhof V., Pasut A., Carmeliet P. (2020). COVID-19: The vasculature unleashed. Nat. Rev. Immunol..

[42] Driggin E., Madhavan M.V., Bikdeli B., Chuich T., Laracy J., Biondi-Zoccai G., Brown T.S., Der Nigoghossian C., Zidar D.A., Haythe J. (2020). Cardiovascular Considerations for Patients, Health Care Workers, and Health Systems During the COVID-19 Pandemic. J. Am. Coll. Cardiol..

